# Stem Cells: The Future Promise of Stem Cells in Healthspan

**DOI:** 10.1093/geroni/igaa057.2663

**Published:** 2020-12-16

**Authors:** Ashley Webb

## Abstract

Tissue specific stem cells are critical for maintenance and repair of tissues and organs throughout life. However, during aging, the functionality of stem cells declines, contributing to tissue decline. This symposium will focus on the mechanisms underlying stem cell aging in various compartments, including muscle, brain and the hematopoietic system.

